# Supplementary material for: The investigation of antibacterial properties of peptides and protein hydrolysates derived from serum of Asian water monitor (Varanus salvator)
Source: PLoS One. 2023 Oct 18;18(10):e0292947. doi: 10.1371/journal.pone.0292947 (PMC10584125; doi:10.1371/journal.pone.0292947)
Supplement: S1 Fig — Model were generated by PyMOL. A) AAIMNWKLCAQLAAFCWGSSFM, B) INHFFCDTPALLKATCS, C) CILPLCGWGTYASTS, D) AMLHTCGTFANTFCS. (PDF) [file pone.0292947.s001.pdf]

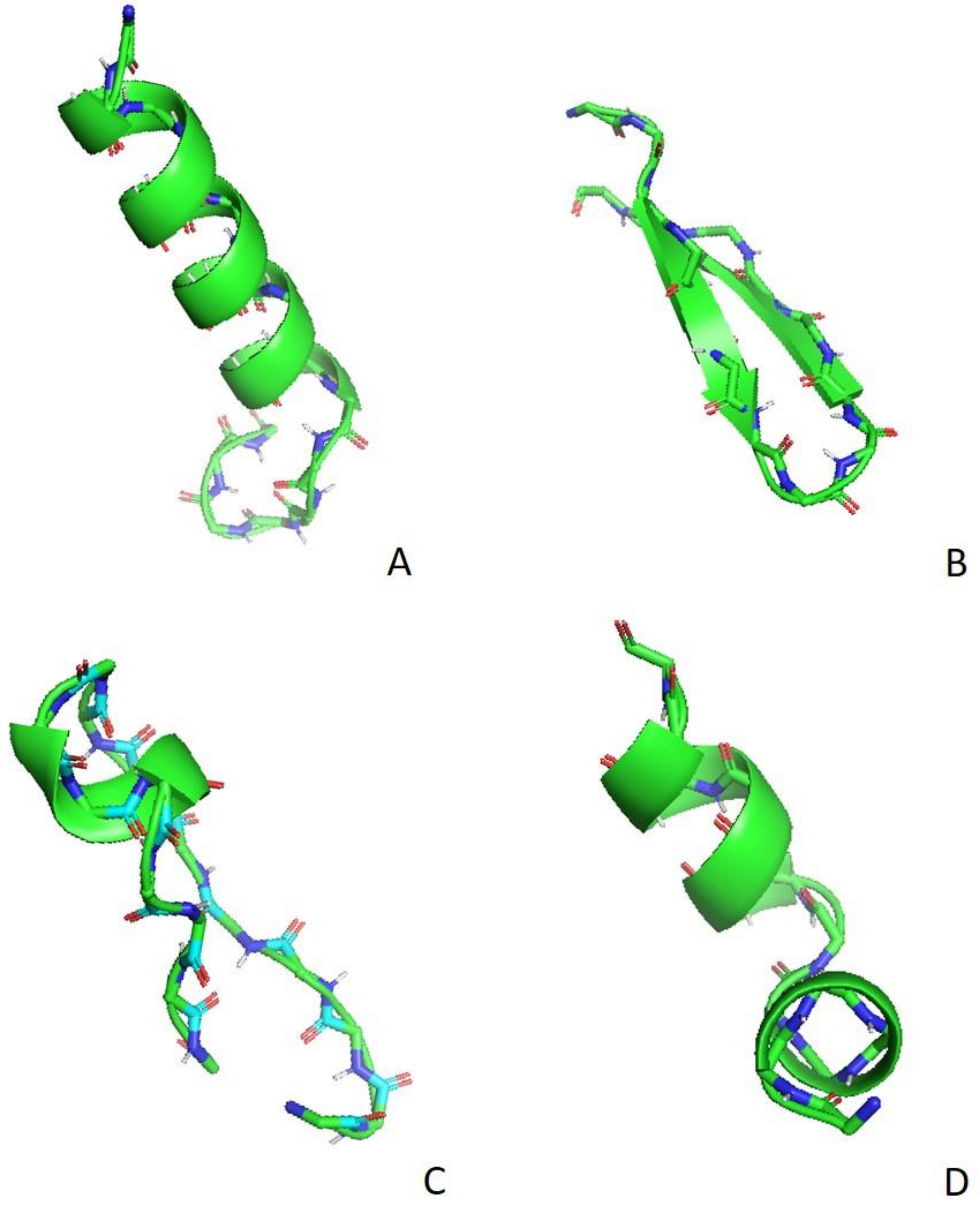

S1 Fig. 3D structures of Antimicrobial peptides from *Varanus salvator* serum were predicted using PEP-FOLD. Model were generated by PyMOL. A) AAIMNWKLCAQLAAFCWGSSFM, B) INHFFCDTPALLKATCS, C) CILPLCGWGTYASTS, D) AMLHTCGTFANTFCS
