# Supplementary material for: The investigation of antibacterial properties of peptides and protein hydrolysates derived from serum of Asian water monitor (Varanus salvator)
Source: PLoS One. 2023 Oct 18;18(10):e0292947. doi: 10.1371/journal.pone.0292947 (PMC10584125; doi:10.1371/journal.pone.0292947)
Supplement: S1 Table — (PDF) [file pone.0292947.s002.pdf]

S1 Table. Inhibitory effect on 10 types of bacteria of crude peptides derived from serum of *Varanus salvator* (n=21).

Varanus salvator	Bacteria									
	Escherichia coli ATCC25922	*Staph. aureus* ATCC25923	MR *Staph. aureus* ATCC43300	*Enterobacter aerogenes* ATCC13048	*Bacillus subtilis* ATCC6633	*Bacillus cereus* ATCC11778	*Acinetobacter baumannii* ATCC19606	*Burkholderia cepacia* ATCC25416	*Pseudomonas aeruginosa* ATCC27853	*Vibrio cholerae* ATCC51394
	---------- % inhibition ----------									
1	7.5	15.5	0.0	10.2	5.3	16.6	19.5	28.9	15.7	0.0
2	0.0	18.0	14.7	35.6	37.9	30.0	53.9	37.4	26.3	0.0
3	9.0	7.6	1.6	11.1	0.0	22.6	34.9	35.4	20.8	0.0
4	12.1	7.2	0.0	17.1	0.0	7.4	11.0	24.6	15.3	0.0
5	10.4	8.9	0.9	21.0	0.0	15.6	20.8	28.1	17.4	0.0
6	15.9	7.2	16.7	24.9	0.0	20.4	28.4	32.1	21.7	0.0
7	18.4	0.0	9.9	18.2	0.0	18.6	24.0	37.2	18.1	0.0
8	7.3	7.3	0.0	10.9	0.0	4.1	8.8	16.6	16.5	0.0
9	0.0	8.6	0.0	16.4	0.0	16.2	33.0	38.2	21.2	0.0
10	1.7	9.6	0.0	5.1	0.0	12.7	7.5	22.1	23.1	0.0
11	0.0	0.5	11.6	34.2	0.0	25.0	46.6	37.7	29.4	1.1
12	0.0	9.2	6.5	38.7	0.0	19.7	54.9	38.0	34.4	0.0
13	0.0	7.0	15.3	33.7	0.0	19.0	48.4	35.3	28.1	0.0
14	0.0	10.0	14.3	29.6	0.0	20.6	46.0	45.7	32.4	0.2
15	0.0	0.0	0.0	25.6	0.0	16.4	31.8	31.5	27.2	0.0
16	3.8	9.3	6.9	40.9	0.0	11.9	44.1	26.9	28.4	0.0
17	0.0	9.4	11.2	32.9	0.0	15.5	46.8	42.4	31.7	0.0
18	0.0	12.2	18.5	32.1	0.0	18.0	50.7	47.7	34.4	1.0
19	0.0	0.0	5.7	33.9	0.0	8.5	43.1	46.0	32.8	0.0
20	11.1	0.0	3.2	34.3	0.0	4.3	33.4	34.0	25.8	0.0
21	0.0	0.0	0.0	12.8	0.0	2.6	24.5	28.5	23.2	0.0
Median	0.0	7.6	5.7	25.6	0.0	16.4	33.4	35.3	25.8	0.0
SD	6.0	5.2	6.6	10.9	8.3	7.1	14.9	8.1	6.3	0.3
