# Supplementary material for: The investigation of antibacterial properties of peptides and protein hydrolysates derived from serum of Asian water monitor (Varanus salvator)
Source: PLoS One. 2023 Oct 18;18(10):e0292947. doi: 10.1371/journal.pone.0292947 (PMC10584125; doi:10.1371/journal.pone.0292947)
Supplement: S2 Table — (PDF) [file pone.0292947.s003.pdf]

S2 Table. Inhibitory effect on 10 types of bacteria of protein hydrolysates derived from serum of *Varanus salvator* (n=21).

Varanus salvator	Bacteria									
	Escherichia coli ATCC25922	*Staph. aureus* ATCC25923	MR *Staph. aureus* ATCC43300	*Enterobacter aerogenes* ATCC13048	*Bacillus subtilis* ATCC6633	*Bacillus cereus* ATCC11778	*Acinetobacter baumannii* ATCC19606	*Burkholderia cepacia* ATCC25416	*Pseudomonas aeruginosa* ATCC27853	*Vibrio cholerae* ATCC51394
	----- % inhibition -----									
1	0.0	0.0	0.0	0.9	0.0	0.0	6.4	23.9	18.2	0.0
2	0.0	0.0	0.0	0.0	0.0	0.0	8.5	23.1	16.2	0.0
3	5.3	0.0	1.0	6.2	0.0	0.0	16.9	24.8	17.9	0.0
4	0.2	0.0	2.0	6.8	0.0	0.0	22.9	27.1	26.5	0.0
5	0.6	0.0	1.9	4.4	0.0	0.0	16.0	34.1	27.2	0.0
6	4.5	0.0	0.0	2.8	0.0	0.0	22.3	33.4	25.0	0.0
7	17.2	10.7	0.0	5.5	0.0	0.0	8.2	9.8	8.5	0.0
8	14.8	13.7	0.0	9.5	0.0	0.0	11.4	13.2	10.4	0.0
9	11.0	20.2	0.0	8.2	0.0	0.0	4.8	22.6	10.6	0.0
10	16.3	5.4	0.0	6.6	0.0	0.0	18.2	13.8	12.5	0.0
11	18.3	10.9	0.0	5.8	0.0	0.0	7.9	8.5	7.9	0.0
12	14.9	18.3	0.0	8.0	0.0	0.0	14.2	18.8	12.0	0.0
13	15.7	14.6	0.0	8.0	0.0	0.0	14.4	31.1	13.4	0.0
14	15.2	2.2	0.0	11.2	0.0	0.0	27.4	30.4	13.2	0.0
15	14.6	0.0	0.0	6.1	0.0	0.0	1.0	12.4	4.9	0.0
16	12.4	6.4	0.0	7.2	0.0	0.0	6.2	24.3	9.5	0.0
17	11.1	2.3	0.0	7.6	0.0	0.0	13.5	19.1	11.9	0.0
18	11.7	0.0	0.0	7.0	0.0	0.0	20.8	30.2	12.5	0.0
19	16.2	0.0	0.0	4.1	0.0	0.0	11.4	24.5	8.2	0.0
20	11.1	4.2	0.0	6.4	0.0	0.0	18.8	27.1	11.1	0.0
21	7.2	0.0	0.0	2.8	0.0	0.0	11.0	31.0	10.8	0.0
Median	11.7	2.2	0.0	6.4	0.0	0.0	13.5	24.3	12.0	0.0
SD	6.2	6.8	0.6	2.7	0.0	0.0	6.8	7.8	6.1	0.0
