# Supplementary material for: The investigation of antibacterial properties of peptides and protein hydrolysates derived from serum of Asian water monitor (Varanus salvator)
Source: PLoS One. 2023 Oct 18;18(10):e0292947. doi: 10.1371/journal.pone.0292947 (PMC10584125; doi:10.1371/journal.pone.0292947)
Supplement: S3 Table — (PDF) [file pone.0292947.s004.pdf]

S3 Table. Amino acid properties of 41 Antimicrobial peptides derived from *Varanus salvator* serum.

Sequence	Hydrophobic (%)	Acidic (%)	Basic (%)	Neutral (%)
AAIMNWKLCAQLAAFCWGSSFM	63.64	0	4.55	31.82
INHFFCDTPALLKATCS	47.06	5.88	11.76	35.29
CILPLCGWGTYASTS	40	0	0	60
AMLHTCGTFANTFCS	40	0	6.67	53.33
CKYKGPSTQGCVLN	21.43	0	14.29	64.29
AVWAFVVCIPFFF	92.31	0	0	7.69
CSFPFIYKGKTYTECTS	23.53	5.88	11.76	58.82
CCLNPILYAF	60	0	0	40
MGLLSTMVGGFGGLN	46.67	0	0	53.33
AHPMPIPAWILMAM	92.86	0	7.14	0
GSTDKSPWCATTSNYDRDRKWKPCA	24	12	20	44
CPVDQTGYRDMRCRN	20	13.33	20	46.67
AFLWFGCLMAF	81.82	0	0	18.18
ACGGWLRRHAI	45.45	0	27.27	27.27
CLPNSACVQTSPG	38.46	0	0	61.54
GSGCGLGSTSGIRDLRNGFCGSGP	20.83	4.17	8.33	66.67
CAINLCPNEPLKYFLVCQYCPG	45.45	4.55	4.55	45.45
DFWSQICSSW	40	10	0	50
FDPLGSARLPFSLHFF	62.5	6.25	12.5	18.75
EYVGWWTPSWVSQGY	40	6.67	0	53.33
CPIQCNAQQTGPWTSAKS	33.33	0	5.56	61.11
AKMGFPFRRG	50	0	30	20
GLWAMVWHHST	54.55	0	18.18	27.27
ACFGFGMVAGP	63.64	0	0	36.36
QTQNLGGFGGVMTS	28.57	0	0	71.43
GGYPCGQPMMPGVYT	40	0	0	60
GGSGNPSHKPRS	16.67	0	25	58.33
CLFFLSAGNAHLNRLLW	58.82	0	11.76	29.41
DLYPTDPCCGYTV	30.77	15.38	0	53.85
APQEYTHYPPPCG	38.46	7.69	7.69	46.15
CQNASVFNTGAAAAAAAH	55.56	0	5.56	38.89
CAPSEPFPVQ	60	10	0	30
CPDARVMLNTTCTSGKS	29.41	5.88	11.76	52.94
CPPFKPDVYNSNI	46.15	7.69	7.69	38.46
AMLCGFWLFAQS	66.67	0	0	33.33
AYPWWHMTDYQLCAGILGGGRDTC	37.5	8.33	8.33	45.83
ERCYQGTSNR	0	10	20	70
ACFSWKKDKDYNATTACWFI	40	10	15	35
DNRPFYVECPS	36.36	18.18	9.09	36.36
AFMAYFYLQTFCHAF	60	0	6.67	33.33
ACMRDSGGPLLC	41.67	8.33	8.33	41.67
