# Supplementary material for: The investigation of antibacterial properties of peptides and protein hydrolysates derived from serum of Asian water monitor (Varanus salvator)
Source: PLoS One. 2023 Oct 18;18(10):e0292947. doi: 10.1371/journal.pone.0292947 (PMC10584125; doi:10.1371/journal.pone.0292947)
Supplement: Attachment — Submitted filename: Comments and review of paper[535].docx [file pone.0292947.s005.docx]

The manuscript titled as “Investigation of Antibacterial Properties of Peptides and Protein Hydrolysates Derived from Serum of Asian Water Monitor (*Varanus salvator*)” represents an interesting study and is well written that only need minor modifications. However, I feel that some relevant images can be added to make the study more attractive and interesting. Please consider the following points:

1. **Title and abstract**:

Title and abstract are accurate and adequately addressed. However, a graphical abstract can be added.

2. **Introduction:**
1. L- 42: Please give the examples of resistant and non-resistant pathogenic bacteria.
2. L-73: ‘evidence of reduced in bacterial growth’ these sentence needs to be re-written.
3. L-80: ‘according to the list of Thailand protected species’, please give an appropriate reference for this sentence.
3. **Materials and Methods**
1. L-27: Why Lowry's method is selected for protein determination over other methods?
2. L-130: Why boiling for 10 minutes is done?
3. L-155: ‘Colistin was used as positive control while hydrolysate buffer was used as negative control’. Please specify reason for this statement.
4. L-156: mention complete formula in mathematical manner.
5. L-169: Specify the reason for the use of nitrogen as a drying gas.
4. **Results:**
1. **Table 2. Antimicrobial peptide prediction of *Varanus salvator* serum, using’** this line seems incomplete.
2. L-247 **‘**5 Gram-negative and 2 Gram-positive bacteria,’ please mention the names of concerned bacteria and also mention reference for this statement.
3. L-269: ‘high isoelectric point’ please mention the numerical value also.
4. L-273 ‘some mechanisms can be proposed’ give brief description of the mechanisms that can be proposed.
5. **Conclusion:**
1. L-294 Antimicrobial peptides…..Hydrophobic, this line is not in flow with this paragraph, so needs to be re-written.
2. Conclusion section is written in brief. Please add more relevant points in it.
3. Also write a concluding line in the end with future scope of this research.
