# Supplementary material for: The investigation of antibacterial properties of peptides and protein hydrolysates derived from serum of Asian water monitor (Varanus salvator)
Source: PLoS One. 2023 Oct 18;18(10):e0292947. doi: 10.1371/journal.pone.0292947 (PMC10584125; doi:10.1371/journal.pone.0292947)
Supplement: Attachment — Submitted filename: Response to Reviewers [Antibacterial_Peptide_Varanus].docx [file pone.0292947.s006.docx]

Dear reviewers

According to some points raised by the academic reviewers to the manuscript entitled “The Investigation of Antibacterial Properties of Peptides and Protein Hydrolysates Derived from Serum of Asian Water Monitor (*Varanus salvator*)”. Please see the responses (the answers in blue) to each point below. Many thanks for all the helpful points.

Your sincerely,

The authors

**Reviewer #1:**

The authors demonstrated that "antimicrobial peptides were predicted which shows highly hydrophobic". It will be interesting to test and check their amyloidogenicity according the last review for AMPs - Int J Mol Sci. 2022 May 13;23(10):5463. doi: 10.3390/ijms23105463.

Line 45. “ceropin” correct to “cecropin”.

Answer: The word was corrected. (L-46)

Lines 59-60. “Moreover, BmKn-2, an animal peptide especially extracted from scorpion venom, has shown the inhibitory effect on bacterial growth [11, 14-16].” I think that BmKn-2 is not discussed in the cited work #15. Please check.

Answer: A cited work was moved from L-61 to L-65.

Line 101. “digestive System” correct to “digestive system”.

Answer: Word was corrected. (L-103)

Line 117. “a plain blood collecting tube” correct to “a plain blood collection tube”.

Answer: It was corrected. (L-119)

Lines 156-157. “The inhibitory percentage was calculated from [(OD control − OD peptide test)/OD control] × 100”. OD control – is it OD for positive or negative control? If this is the optical density for the negative control, then Table 1 should indicate the antimicrobial efficacy of the crude peptides and protein hydrolysates in comparison with the antibiotic, colistin.

Answer:

Colistin was used as a positive control because it is a peptide antibiotic and it must exhibit antimicrobial activity. 0.5 M sodium acetate was used as buffer for hydrolysis of proteins and it should not show any antimicrobial activity.

The formula is complete as follows. The inhibitory percentage was calculated from [(OD control − OD peptide test)/OD control] × 100 where OD control is the absorbance of control and OD peptide test is the absorbance of the sample. (L-159)

**Reviewer #2:**

The manuscript titled as “Investigation of Antibacterial Properties of Peptides and Protein Hydrolysates Derived from Serum of Asian Water Monitor (*Varanus salvator*)” represents an interesting study and is well written that only need minor modifications. However, I feel that some relevant images can be added to make the study more attractive and interesting. Please consider the following points:

1. **Title and abstract**:

Title and abstract are accurate and adequately addressed. However, a graphical abstract can be added.

Answer:	A missing part has been added in abstract. (L-28)

A graphical abstract was created in a separated file (optional).

2. **Introduction:**
1. L- 42: Please give the examples of resistant and non-resistant pathogenic bacteria.

Answer: The examples were added. (L-42)

2. L-73: ‘evidence of reduced in bacterial growth’ these sentence needs to be re-written.

Answer: The sentence was rewritten. ‘showed evidence of reduction in bacterial growth’ (L-73)

3. L-80: ‘according to the list of Thailand protected species’, please give an appropriate reference for this sentence.

Answer: The sentence was edited and a reference was added. (L-80)

3. **Materials and Methods**
1. L-127: Why Lowry's method is selected for protein determination over other methods?

Answer: Lowry’s method is less sensitive to interfering agents.

2. L-130: Why boiling for 10 minutes is done?

Answer: Boiling the protein hydrolysate can inactivate pepsin (or other proteases) enzyme. (L-132)

3. L-155: ‘Colistin was used as positive control while hydrolysate buffer was used as negative control’. Please specify reason for this statement.

Answer: Colistin was used as a positive control because it is a peptide antibiotic and it must exhibit antimicrobial activity. 0.5 M sodium acetate was used as buffer for hydrolysis of proteins and it should not show any antimicrobial activity.

4. L-156: mention complete formula in mathematical manner.

Answer: The formula is complete as follows. The inhibitory percentage was calculated from [(OD control − OD peptide test)/OD control] × 100 where OD control is the absorbance of control and OD peptide test is the absorbance of the sample. (L-159)

5. L-169: Specify the reason for the use of nitrogen as a drying gas.

Answer: Nitrogen is used as a drying gas in the LC-MS to facilitate buffer desolvation, to enhance sensitivity, and to prevent the entry of undesirable ions into the mass spectrometer.

4. **Results:**
1. **Table 2. Antimicrobial peptide prediction of *Varanus salvator* serum, using’** this line seems incomplete.

Answer: Antimicrobial Peptide Scanner vr.2 (L-237)

2. L-247 **‘**5 Gram-negative and 2 Gram-positive bacteria,’ please mention the names of concerned bacteria and also mention reference for this statement.

Answer: Bacterial names were added. The reference has been placed. (L-250)

3. L-269: ‘high isoelectric point’ please mention the numerical value also.

Answer: “PI > 8” was added. (L-274)

4. L-273 ‘some mechanisms can be proposed’ give brief description of the mechanisms that can be proposed.

Answer: Delete this sentence, because the next sentence is an explanation. (L-278)

5. **Conclusion:**
1. L-294 Antimicrobial peptides…..Hydrophobic, this line is not in flow with this paragraph, so needs to be re-written.

Answer: New sentences have been rewritten. (L-301)

2. Conclusion section is written in brief. Please add more relevant points in it.

Answer: The whole conclusions were customized for more relevant. (L-298)

3. Also write a concluding line in the end with future scope of this research.

Answer: Some future study guidelines have been proposed. (L-307)
