# Supplementary material for: The investigation of antibacterial properties of peptides and protein hydrolysates derived from serum of Asian water monitor (Varanus salvator)
Source: PLoS One. 2023 Oct 18;18(10):e0292947. doi: 10.1371/journal.pone.0292947 (PMC10584125; doi:10.1371/journal.pone.0292947)
Supplement: Attachment — Submitted filename: Response to reviewer #1.docx [file pone.0292947.s007.docx]

**Reviewer #1:**

The authors demonstrated that "antimicrobial peptides were predicted which shows highly hydrophobic". It will be interesting to test and check their amyloidogenicity according the last review for AMPs - Int J Mol Sci. 2022 May 13;23(10):5463. doi: 10.3390/ijms23105463.

**Response from authors**

Based on the reviews you mentioned [1], we had run for amyloidogenic antimicrobial peptide (AAMP) prediction on the AMPs presented in Table 2, using iAMY-SCM : http://camt. pythonanywhere.com/iAMY-SCM at threshold = 288.5625 [2, 3]. We found that 14 sequences of 41 AMPs were showed AAMP properties, however, only 5 AAMPs were characterized to be the peptides originated from *Varanus spp*., including NADH dehydrogenase subunit 2 (mitochondrion); olfactory receptor, partial; olfactory receptor 1052-like; kallikrein-Vgou1 and ESP-Vind2, partial. The rest are uncharacterized proteins or belonged to other species such as bacteria and virus.

Since we used peptides with low MW (3 kDa or smaller), therefore, the results of polymerized AAMP could not be tested in this experiment. However, the mechanism of action of predicted AAMP is interesting for further in-depth study.

**References**

1.	Galzitskaya OV, Kurpe SR, Panfilov AV, Glyakina AV, Grishin SY, Kochetov AP, et al. Amyloidogenic Peptides: New Class of Antimicrobial Peptides with the Novel Mechanism of Activity. International Journal of Molecular Sciences [Internet]. 2022; 23(10).

2.	Teng Z, Zhang Z, Tian Z, Li Y, Wang G. ReRF-Pred: predicting amyloidogenic regions of proteins based on their pseudo amino acid composition and tripeptide composition. BMC Bioinformatics. 2021;22(1):545. doi: 10.1186/s12859-021-04446-4.

3.	Charoenkwan P, Kanthawong S, Nantasenamat C, Hasan MM, Shoombuatong W. iAMY-SCM: Improved prediction and analysis of amyloid proteins using a scoring card method with propensity scores of dipeptides. Genomics. 2021;113(1, Part 2):689-98. doi: https://doi.org/10.1016/j.ygeno.2020.09.065.
